# Tar Smoke for Diphtheria

**Published:** 1884-07

**Authors:** 


### TAR SMOKE FOR DIPHTHERIA.

RUTH Lockwood, the nine-year-old child of Thomas Lockwood, a compositor in the *Times* office, became violently ill with diphtheria on Tuesday night. She was so weak that it was deemed dangerous to try tracheotomy, or cutting open the windpipe. On Thursday Dr. Nichols of 117 West Washington place, who was attending her, received a copy of the Paris *Figaro*, which contained a report made to the French Academy of Medicine by Dr. Delthill. Dr. Delthill said that the vapors of liquid tar and turpentine would dissolve the fibrinous exudations which choke up the throat in croup and diphtheria.

Dr. Delthill's process was described. He pours equal parts of turpentine and liquid tar into a tin pan or cup and sets fire to the mixture. A dense resinous smoke arises, which obscures the air of the room.

"The patient," Dr. Delthill says, "immediately seems to experience relief; the choking and rattle stop; the patient falls into a slumber and seems to inhale the smoke with pleasure. The fibrinous membrane soon becomes detached, and the patient coughs up microbicides. These, when caught in a glass, may be seen to dissolve in the smoke. In the course of three days afterward the patient entirely recovers."

Dr. Nichols tried this treatment yesterday with little Ruth Lockwood. She was lying gasping for breath when he visited her. First pouring about two tablespoonfuls of liquefied tar on an iron pan, he poured as much turpentine over it and set it on fire. The rich resinous smoke which rose to the ceiling was by no means unpleasant. As it filled the room the child's breathing became natural, and as the smoke grew dense she fell asleep.

